# The Immunological Intersection of Psoriasis and Autoimmune Diseases: Shared Pathways and Clinical Implications

**DOI:** 10.3390/life16091542

**Published:** 2026-09-16

**Authors:** Alexandra-Petruța Savu, Rodica Olteanu, Diana Mihaela Ciuc, Maria-Alexandra Tiron, Traian-Vasile Constantin, Costina-Cristiana Mutu, Ștefana Bucur, Maria-Magdalena Constantin

**Affiliations:** 1 Faculty of Medicine, “Carol Davila” University of Medicine and Pharmacy, 050474 Bucharest, Romania; diana_ciuc@yahoo.com (D.M.C.); traianc29@yahoo.com (T.-V.C.); cristiana.terziu@gmail.com (C.-C.M.); stefana.bucur@umfcd.ro (Ș.B.); maria.constantin@umfcd.ro (M.-M.C.); 2 Department of Dermatology, CF2 Clinical Hospital, 011464 Bucharest, Romania; maria.tiron0125@rez.umfcd.ro; 3 2nd Department of Dermatology, Colentina Clinical Hospital, 020125 Bucharest, Romania; 4 Faculty of Medicine, Titu Maiorescu University, 040051 Bucharest, Romania; 5 Otolaryngology Department, CF2 Clinical Hospital, 011464 Bucharest, Romania; 6 Department of Urology, “Professor Doctor Theodor Burghele” Clinical Hospital, 061344 Bucharest, Romania

**Keywords:** psoriasis, autoimmunity, inflammation

## Abstract

Psoriasis is a chronic inflammatory disease associated with multiple autoimmune conditions. This article reviews the relationship between psoriasis and autoimmune blistering dermatoses, vitiligo, alopecia areata, autoimmune thyroid disorders, systemic lupus erythematosus, and celiac disease, highlighting shared genetic susceptibility and overlapping immune pathways. The coexistence of these conditions may influence clinical presentations, prognosis, and treatment selection. In addition, various psoriasis therapies have been associated with the onset or exacerbation of concomitant autoimmune disorders. Therefore, recognizing these conditions is essential for individualized management and a multidisciplinary approach.

## 1. Introduction

Psoriasis is a common inflammatory skin disorder associated with other chronic conditions and affects both adults and children [1,2]. It is a multifactorial disease caused by the interaction between inherited susceptibility alleles and environmental triggers, such as stress, trauma, infection (e.g., streptococcal, human immunodeficiency virus), obesity, smoking, alcohol consumption, and exposure to certain drugs [1,3,4,5,6,7].

The pathophysiological pathways consist of a complex interplay between innate and adaptive immunity, resulting in uncontrolled proliferation and differentiation of keratinocytes, acanthosis, neovascularization, and inflammatory infiltrates in the skin [3,8]. An important role in the development of psoriasis is mediated by the dysregulation of both cutaneous and intestinal microbiomes [8,9]. Regarding the predisposing genetic factor, the risk of developing psoriasis is two to three times higher in monozygotic twins than in dizygotic twins. A positive family history is found in more than 20% of patients [1,6,10,11].

For the purposes of this review, a literature search was performed using the PubMed and Web of Science databases. Search terms included psoriasis, autoimmune disease, autoimmunity, autoimmune blistering dermatoses, bullous pemphigoid, pemphigus, vitiligo, alopecia areata, autoimmune thyroid disease, systemic lupus erythematosus, celiac disease, IL-23, IL-17, Th17, biologic therapies, and JAK inhibitors. The literature search included articles published up to June 2026. First, we evaluated the titles and abstracts of the articles, followed by full-text assessment of potentially eligible articles. Original studies, systematic reviews, meta-analyses, narrative reviews, case series, and relevant case reports published in English were considered for inclusion based on their relevance to the scope of this review.

## 2. The Immunopathogenesis of Psoriasis and Its Association with Autoimmune Diseases

A recent multi-ancestry meta-analysis of genome-wide association studies (GWAS) identified 74 genome-wide significant loci associated with psoriasis, 32 of which were novel psoriasis risk loci. Genetic correlation analyses showed that psoriasis is positively correlated with autoimmune diseases such as ulcerative colitis, Crohn’s disease, rheumatoid arthritis, and type 1 diabetes [12].

Psoriasis is a T-cell-mediated disease. The onset of cutaneous lesions is due to numerous environmental factors that activate plasmacytoid dendritic cells (pDCs), a unique subset of dendritic cells that is increased in early psoriatic lesions [13,14]. pDCs are triggered by molecules released from damaged keratinocytes, such as antimicrobial peptides (AMPs). Activated pDCs produce type 1 interferons (IFN-α and IFN-β), which along with tumor necrosis factor alpha (TNF-α) (produced by damaged keratinocytes) stimulate the pDCs to differentiate into myeloid dendritic cells (mDCs). mDCs secrete TNF-α, IL-12, and IL-23, leading to T-cell differentiation toward Th1, Th17, and Th22 subsets in the adaptive immune response [15,16]. Th1 cells produce IFN-γ and TNF-α, with proinflammatory effects; Th17 cells produce IL-17, especially IL-17A and IL-17F, as well as IL-22, leading to keratinocyte activation and proliferation, neutrophil recruitment and angiogenesis; Th22 cells produce IL-22, maintaining the inflammatory process [16,17,18].

The role of keratinocytes as triggering factors in psoriasis remains a subject of debate. Keratinocytes secrete antimicrobial peptides (AMPs), such as β-defensins, cathelicidins, and S100 proteins, in response to trauma. Cathelicidin LL-37 can activate Toll-like receptors (TLRs), thereby inducing IFN-α production by pDCs and contributing to cutaneous inflammation [19,20]. LL-37 also acts as an autoantigen by stimulating CD4+ and CD8+ T cells [21]. Similarly, ADAMTS-like protein 5 (ADAMTSL5) produced by epidermal melanocytes may act as an autoantigen. In psoriasis, IL-17 stimulates keratinocytes to generate ADAMTSL5 [22]. Cytokines and chemokines produced by keratinocytes contribute to the amplification of the inflammatory cascade.

Current evidence supports the pathogenic triad of dendritic cells, Th17 cells, and keratinocytes, in which the IL-23/Th17 axis plays a central role in the persistence of psoriatic inflammation [23]. The central role of this axis in psoriasis pathogenesis has contributed to the development of selective biologic therapies, such as anti-IL-17 and anti-IL-23 monoclonal antibodies [24]. The clinical relevance of the IL-23/Th17 axis in psoriatic disease is supported by recent real-world evidence from a multicenter observational study of patients with psoriatic arthritis treated with guselkumab, which demonstrated improvements in both articular and cutaneous disease [25].

Psoriasis has been linked to both autoimmune dermatological conditions, including autoimmune bullous skin diseases, vitiligo, and alopecia areata, and systemic autoimmune disorders, such as inflammatory bowel disease, celiac disease, autoimmune thyroid diseases, multiple sclerosis, and systemic lupus erythematosus [26,27].

This review examines the connection between psoriasis and associated autoimmune diseases, particularly dermatological and systemic conditions, focusing on shared genetic and immunopathogenic pathways, as well as treatment approaches for patients presenting with these coexisting disorders.

## 3. Autoimmune Dermatological Diseases Associated with Psoriasis Vulgaris

### 3.1. Association of Psoriasis Vulgaris with Autoimmune Blistering Dermatoses

Autoimmune blistering dermatoses represent a group of rare, potentially life-threatening disorders caused by IgG autoantibodies directed against structural proteins of the skin and mucous membranes. These autoantibodies either target desmosomal proteins, leading to the pemphigus spectrum characterized by fragile intraepidermal blisters and erosions, or hemidesmosomal proteins, resulting in the pemphigoid spectrum, which presents with tense subepidermal blisters [28,29]. In the general population, pemphigus vulgaris is the most common form of pemphigus, followed by pemphigus foliaceus [28,29]. The incidence of pemphigus ranges from 0.76 to 16.1 cases per million per year [30,31]. It typically affects individuals between 45 and 65 years of age, although it may occur at any age, and shows a slight male predominance [32].

A retrospective cohort study reported a higher prevalence of pemphigus among patients with psoriasis compared to controls (OR 48.1) [33]. More recent data from another retrospective cohort study demonstrated an approximately threefold increased risk of developing pemphigus in patients with psoriasis (HR 3.25; 95% CI, 1.70–6.21), particularly among women (HR 4.20) [34]. Available case series indicate that pemphigus vulgaris and pemphigus foliaceus (including pemphigus erythematosus) are the most commonly reported subtypes associated with psoriasis [35,36,37]. However, in the absence of prospective studies, the true risk of pemphigus development during the course of psoriasis vulgaris remains uncertain.

Bullous pemphigoid is the most common autoimmune blistering disease, with an incidence ranging from 2.4 to 23 cases per million per year. In contrast to most autoimmune diseases, its incidence increases exponentially with age, predominantly affecting individuals older than 70 years [38]. Epidemiological data indicate a higher incidence in women up to the age of 75, followed by a reversal of the sex ratio, with a higher incidence in men thereafter [39,40,41]. Other conditions within the pemphigoid spectrum include mucous membrane pemphigoid (cicatricial), anti-laminin-γ1 pemphigoid (formerly known as anti-p200 pemphigoid), linear IgA dermatosis, and epidermolysis bullosa acquisita. Notably, the first descriptions of anti-laminin-γ1 pemphigoid were reported in patients with psoriasis who exhibited autoantibodies against a previously unidentified antigen, and approximately one-third of subsequent cases have been associated with psoriasis [42].

A retrospective cohort study demonstrated that patients with psoriasis have a significantly higher risk of developing pemphigoid compared to the general population (OR 14.8; 95% CI, 5.00–43.50; *p* < 0.0001) [33]. Although the association between psoriasis vulgaris and pemphigoid-spectrum disorders is estimated to be approximately three times more frequent than that with pemphigus-spectrum disorders, it remains relatively rare [36]. In line with findings from a case series including 145 Japanese patients, psoriasis was most frequently associated with bullous pemphigoid (63%), followed by anti-laminin-γ1 pemphigoid (37%), their combination (8%), linear IgA dermatosis (2.07%), and epidermolysis bullosa acquisita (1.38%) [36,37].

In a retrospective cohort study conducted in Taiwan, the mean time to the diagnosis of bullous pemphigoid in patients with psoriasis was 2.86 years, with a significant proportion of cases occurring within the first year, followed by a subsequent decline in incidence. In the same study, 17.1% of patients with both psoriasis and bullous pemphigoid were under 60 years of age, which differs from the typical age distribution of bullous pemphigoid [43]. Similar findings regarding the mean diagnosis interval were reported in another case–control study from Taiwan [44]. However, in a German case series including 40 patients with psoriasis, the mean diagnostic interval was 20 years [45]. Likewise, a case–control study from Israel reported a mean diagnostic interval of 25.2 ± 18.6 years [46]. These discrepancies may reflect differences in study populations, demographic characteristics, diagnostic criteria, treatment exposure, potential confounding factors, and study design. For instance, in Caucasian populations, the incidence of psoriasis vulgaris tends to decrease with age, whereas the incidence of bullous pemphigoid increases, potentially accounting for the longer interval observed in the German cohort [43]. Further studies are required to clarify the temporal relationship between psoriasis vulgaris and bullous pemphigoid.

Some authors have suggested a potential link between early-onset psoriasis and pemphigus through shared HLA class II alleles, such as DRB1 and DQB1 [47,48]. In contrast, current evidence suggests that psoriasis and pemphigoid do not share a common genetic susceptibility. It is more likely that epigenetic modifications within psoriatic lesions contribute to the development of these conditions, particularly pemphigoid [34,36,49]. Chronic inflammation in psoriasis may lead to structural alterations in desmosomal and hemidesmosomal proteins, resulting in the exposure of previously hidden epitopes to the immune system. This process, known as “epitope spreading,” ultimately triggers a secondary humoral immune response [50,51]. Supporting this hypothesis, blistering lesions have been reported to preferentially develop on psoriatic plaques (an inverse Koebner phenomenon) [52,53,54,55]. Another study used this mechanism to explain the presence of basement membrane autoantibodies in patients with psoriasis without clinically evident blistering disease [35]. Notably, in most reported cases, psoriasis preceded the onset of pemphigus, with no available data supporting the reverse association [53]. However, in a case series including 62 patients with pemphigoid, 7 patients subsequently developed psoriasis, suggesting the involvement of additional pathogenic mechanisms [56].

It has been observed that lesional skin of patients with bullous pemphigoid contains a significant population of IL-17-secreting CD4+ T lymphocytes, and that serum levels of IL-17 are elevated compared to control groups [57]. Given that the pathogenesis of psoriasis vulgaris is largely driven by the IL-23/Th17/IL-17 axis, these findings suggest an immunological link between the two conditions, although they do not explain the typical temporal sequence of disease onset [36]. Nevertheless, in cases where pemphigus or pemphigoid develop during the course of psoriasis, a potential shift in the immune response from a Th1-dominant profile toward a Th2-dominant profile may favour autoantibody production [58]. Furthermore, dysregulation of T-cell responses, leading to reduced immune tolerance and subsequent increased autoantibody production, has been proposed as a mechanism underlying the coexistence of these conditions in certain individuals [59].

In genetically susceptible individuals, certain systemic or topical medications have been proposed to interfere with the antigenic properties of desmosomal and hemidesmosomal proteins, potentially triggering autoantibody production [60]. A systematic review has identified several therapies within this category, including antipsoriatic treatments such as topical corticosteroids, anthralin, coal tar, and TNF-α inhibitors (etanercept, adalimumab, infliximab), as well as medications commonly used for psoriasis-associated comorbidities, including gliptins, loop and thiazide diuretics, and nonsteroidal anti-inflammatory drugs (NSAIDs) [60,61]. Authors of a systematic review on bullous pemphigoid induced by biologic therapies used in psoriasis suggest that these agents may disrupt immune homeostasis rather than produce a purely inhibitory effect on cytokine pathways [62]. Similarly, phototherapy has been reported as a potential trigger for both pemphigus and pemphigoid [63]. This association has been more frequently reported in bullous pemphigoid, particularly with PUVA therapy, and may result from basal keratinocyte injury leading to altered antigenicity [51,64]. In the case of pemphigus, ultraviolet radiation has been shown to induce epidermal instability and even acantholysis in clinically unaffected skin [34].

Taken together, the management of psoriasis in patients with coexisting autoimmune blistering dermatoses remains challenging due to the complex and sometimes paradoxical effects of available therapies.

### 3.2. Association Between Psoriasis and Vitiligo

Vitiligo is the most common depigmenting disorder of the skin and mucous membranes and is caused by the selective loss of melanocytes [65]. Clinically, it is characterized by the appearance of well-demarcated, chalk-white macules of varying sizes, without scaling, and with a significant psychosocial impact [65,66]. Depending on the distribution of these lesions, vitiligo is classified into two main forms, nonsegmental (the majority of cases) and segmental, with mixed forms also described [67,68].

Vitiligo affects all skin phototypes, with a prevalence in the general population estimated at 0.5–2%. Although there are no significant sex differences in incidence, women tend to seek medical attention more frequently, likely due to the greater psychosocial burden [65,66]. Nonsegmental vitiligo may occur at any age but typically develops between 10 and 30 years, with nearly 50% of cases occurring before the age of 20, whereas segmental vitiligo tends to have an earlier onset [66].

The association between psoriasis and vitiligo has been recognized since 1890; however, the relationship between the two conditions remains uncertain [69]. A systematic review published in 2012, which included 35 case reports and 7 case series, found that the frequency of coexistence of the two diseases was consistent with the expected prevalence in the general population, suggesting a coincidental association rather than a true increased risk [70].

However, familial aggregation observed in both psoriasis and vitiligo supports the presence of a shared polygenic autoimmune background, suggesting that their coexistence may reflect common genetic susceptibility rather than a purely coincidental association [71]. A study conducted in China showed that genetic variants within the HLA-C/HLA-B region are associated with both psoriasis and vitiligo, providing important evidence that these two conditions share a common susceptibility locus within the major histocompatibility complex (MHC) and suggesting a shared molecular background [72]. Moreover, a meta-analysis published in 2019, which included seven studies evaluating the risk of vitiligo in patients with psoriasis [33,71,73,74,75,76,77], and four studies assessing the risk of psoriasis in patients with vitiligo [44,71,78,79], reported an approximately twofold increased risk of developing vitiligo among patients with psoriasis (pooled OR 2.29; 95% CI 1.56–3.37). Notably, substantial statistical heterogeneity was observed among the included studies. Similarly, an approximately threefold increased risk of psoriasis was identified in patients with vitiligo (pooled OR 3.43; 95% CI 1.86–6.33), although these results should be interpreted with caution due to the high heterogeneity and potential bias related to insufficient adjustment for confounding variables [80].

A retrospective cohort study published in 2022 demonstrated that patients with vitiligo have a significantly increased risk of developing psoriasis (fully adjusted HR 1.71; 95% CI, 1.35–2.17; *p* < 0.001), whereas patients with psoriasis showed only a borderline increased risk of developing vitiligo (fully adjusted OR 1.19; 95% CI 1.01–1.40; *p* = 0.051). Furthermore, patients with vitiligo and concomitant psoriasis tended to have a later disease onset and more frequently presented metabolic and cardiovascular comorbidities compared to controls [81].

More recent data published in 2024 reported that, among 73 patients from China, 69.4% had vitiligo as the initial diagnosis, whereas only 30.6% were first diagnosed with psoriasis [69]. Similar trends have been observed in cohorts from Korea and Israel, suggesting that vitiligo more commonly precedes psoriasis and may create a permissive environment for its development [81,82]. In the previously mentioned review including 36 articles, lesions of vitiligo and psoriasis overlapped in 18 cases, were independent in 2 cases, and showed a mixed distribution pattern in 11 cases [70]. Both conditions are known to exhibit the Koebner phenomenon; therefore, when lesions coexist within the same anatomical area, reciprocal triggering may occur [83].

The first study employing self-controlled transcriptomic sequencing of biopsy samples demonstrated that the gene expression profile of skin affected by both vitiligo and psoriasis more closely resembles that of psoriatic skin. Based on these findings, the authors suggested that, in patients with both conditions, psoriasis may develop on a background of pre-existing vitiligo [69]. Consistent with this observation, immunohistochemical analyses of biopsy specimens from patients with both diseases revealed increased expression of CD4, CD8, Foxp3, and IL-17A [84].

Although immune activation differs between the two conditions, their pathogenesis appears to share several similarities, including Th1/Th17 polarization, defective regulatory T-cell function, activation of Langerhans cells, involvement of CD8+ T lymphocytes, and persistence of tissue-resident memory T cells [85,86]. Additional evidence supporting a shared pathogenic background includes the observation that IFN-α therapy for hepatitis C may induce both psoriasis and vitiligo [87,88,89,90]. Furthermore, Janus kinase (JAK) inhibitors such as tofacitinib have been reported as potential therapeutic options in both psoriasis and vitiligo, while narrowband UVB phototherapy has also been associated with clinical improvement in both conditions [91,92,93].

Interestingly, several cases of new-onset vitiligo temporally associated with biologic therapy in patients with psoriasis have been reported in the literature. However, improvement of both psoriasis and pre-existing vitiligo following the initiation of biologic treatment has also been described, highlighting the complexity of this relationship. According to a case report and narrative review published in 2021, anti-TNF-α agents are most frequently associated with the onset or exacerbation of vitiligo, whereas anti-IL-17 and anti-IL-12/23 are more commonly associated with progression of pre-existing vitiligo. To date, no association with anti-IL-23 agents has been reported in the literature [94]. The available evidence is limited and does not establish a causal relationship between biologic therapy and the onset or worsening of vitiligo. The exact mechanism underlying this association remains unclear; however, it has been hypothesized that biologic therapies may induce a cytokine imbalance, thereby creating a permissive environment for the development of vitiligo [95]. Therefore, individualized treatment strategies and close clinical monitoring are essential, and further studies are needed to clarify the optimal therapeutic approach in this patient population.

### 3.3. Association of Psoriasis with Alopecia Areata

Alopecia areata is an autoimmune disorder characterized by circumscribed and symptomless patches of non-scarring hair loss involving the scalp and other hair-bearing areas such as the beard [96]. Alopecia areata presents with several clinical variants and may progress to more extensive forms such as alopecia totalis or alopecia universalis [97]. Alopecia areata affects approximately 2% of the global population and may present at any age, although it appears to be more prevalent in children than in adults [98,99]. It is more frequent in women than in men, especially in cases of late-onset disease, defined as onset beyond 50 years of age [100].

Psoriasis has been associated with an increased risk of alopecia areata, with odds ratios ranging from approximately 2.5 to 4.7 in population-based studies, indicating a moderate to strong association between the two conditions [33,73]. A meta-analysis published in 2022, comprising 27 studies, found that the pooled prevalence of alopecia areata among individuals with psoriasis was 0.5% (95% CI 0.3–0.7%). The same study reported that psoriasis was associated with an increased likelihood of alopecia areata (OR 2.71; 95% CI 2.29–3.21), whereas alopecia areata was linked to both a higher prevalence (2.5%; 95% CI 2.0–3.0%) and an elevated risk of psoriasis (OR 3.52; 95% CI 1.27–9.74), supporting a bidirectional association between the two diseases [101]. Available evidence suggests that the association between psoriasis and alopecia areata is largely independent of age and sex, as it remains significant across subgroup analyses [101]. Current evidence does not clearly indicate that their coexistence significantly alters the clinical course or prognosis of either condition [96,102].

The association of both psoriasis and alopecia areata with loci within the HLA region of the major histocompatibility complex (MHC), together with evidence of familial aggregation, highlights a common genetic susceptibility underlying these conditions [103]. Similar to vitiligo, alopecia areata is associated with an increased risk of atopy, thyroid disease, or other autoimmune disorders; therefore, screening for these conditions should be considered [97].

Although the exact pathogenic mechanisms are not fully understood, both diseases involve aberrant immune activation with dysregulation of Th1 and Th17 pathways and overlapping cytokine profiles, including IFN-γ, TNF-α, and IL-17. However, alopecia areata is characterized by collapse of hair follicle immune privilege and a T-cell-mediated response against poorly defined follicular autoantigens, whereas psoriasis is driven by dysregulated cutaneous immune responses to identified autoantigens such as LL-37 [48,97]. Interestingly, anti-TNF-α agents, which are widely used in psoriasis, have been reported to induce or exacerbate alopecia areata, highlighting the complex role of cytokine signalling in disease pathogenesis. However, the available evidence does not establish a causal relationship [97,104]. Another important pathogenic pathway in both conditions is the JAK-STAT signalling pathway, which explains why both may benefit from treatment with tofacitinib [105,106].

An interesting clinical feature observed in both alopecia areata and psoriasis is nail pitting, which in alopecia areata typically presents as shallow pits in a regular, grid-like distribution, in contrast to the deeper and more irregular pits seen in psoriasis [97]. In both conditions, nail pitting represents focal defects in keratinization due to the inflammation of the nail matrix. In psoriasis, this is driven by parakeratosis, whereas in alopecia areata it is thought to reflect a similar autoimmune process affecting nail matrix keratinocytes [97,107].

Finally, it is important to differentiate between psoriatic involvement of the scalp, which may lead to hair loss (most commonly through telogen effluvium), and alopecia areata, as management strategies may differ [108]. However, anthralin, a traditional therapeutic agent used in psoriasis, can also be used in alopecia areata to induce contact dermatitis as an immune-deviation strategy in chronic, relapsing cases [97].

## 4. Systemic Autoimmune Diseases Associated with Psoriasis

### 4.1. Association of Psoriasis with Thyroid Autoimmunity

Autoimmune thyroid diseases (AITD) represent a wide spectrum of disorders characterized by an autoimmune response directed against thyroid antigens and predominantly affect women. Graves’ disease and Hashimoto’s thyroiditis represent the most common forms. Thyroid autoimmunity refers to the presence of antibodies directed against thyroid antigens, with or without clinical significance [109].

Hashimoto’s thyroiditis (HT), also known as autoimmune thyroiditis, is a chronic autoimmune disorder characterized by lymphocytic infiltration and progressive destruction of the thyroid gland, often leading to hypothyroidism. The presence of anti-thyroid peroxidase (anti-TPO) and anti-thyroglobulin (anti-Tg) antibodies represents a hallmark of the autoimmune component of the disease but they do not play a direct role in the destruction of the thyroid gland [110]. It is estimated that HT affects 2% of the general population, being more common in older individuals and ten times more frequent in women than in men [111]. Graves’ disease, on the other hand, is a chronic autoimmune disorder characterized by the presence of thyroid-stimulating hormone receptor antibodies (TRAb) that bind to thyroid follicular cells, leading to goitre and usually to hyperthyroidism. These antibodies also target fibroblasts in orbital and dermal tissues, contributing to extrathyroidal manifestations such as Graves’ ophthalmopathy and dermopathy. Graves’ disease is thought to be the most common cause of hyperthyroidism in iodine-sufficient areas, typically affecting middle-aged women [109,112]. In both conditions, serum levels of thyroid hormones (free triiodothyronine, FT3, and free thyroxine, FT4) and thyroid-stimulating hormone (TSH) may fluctuate depending on disease stage and therapeutic interventions [113].

Multiple studies have shown that psoriasis is associated with an increased risk of AITD [114,115]. Although findings remain inconsistent, female sex, obesity, late-onset psoriasis, and psoriatic arthritis have been proposed as potential factors associated with a higher prevalence of thyroid autoimmunity [115]. Despite evidence suggesting that erythrodermic psoriasis is associated with the highest prevalence of thyroid involvement, most studies have not demonstrated a correlation between psoriasis severity and thyroid dysfunction [113].

It has been observed that psoriasis is significantly associated with hypothyroidism (OR 1.34; 95% CI 1.16–1.54), particularly in its subclinical form, and less frequently with hyperthyroidism (OR 1.17; 95% CI 1.03–1.32) [113,116]. A meta-analysis published in 2022, which compared the types of AITD associated with psoriasis, found that HT was by far the most frequent (OR 1.88, 95% CI 1.50–2.35) [117]. Supporting this finding, patients with psoriasis have been reported to exhibit a significantly higher prevalence of thyroid autoimmunity markers, including anti-TPO and anti-Tg antibodies, as well as ultrasound features suggestive of HT, such as hypoechogenicity, pseudo-nodularity, and increased vascularity. However, no significant differences in the prevalence of overt or subclinical hypothyroidism were observed compared with controls, suggesting that psoriasis may be associated with early autoimmune thyroid changes rather than established thyroid dysfunction [118].

Excess thyroid hormone levels may contribute to psoriasis exacerbation by promoting keratinocyte proliferation and modulating inflammatory signalling pathways, whereas the influence of hypothyroidism appears less direct and may be more closely related to shared autoimmune mechanisms than to reduced thyroid hormone levels themselves. Further evidence supporting a link between thyroid function and psoriasis activity comes from reports showing improvement of psoriatic lesions following thyroid-targeted interventions, including propylthiouracil treatment and thyroidectomy [115]. However, some clinical observations suggest that thyroid hormone alterations may also influence skin manifestations, as improvement of psoriasiform lesions has been reported following normalization of thyroxine levels after thyroid hormone replacement therapy in a patient with severe hypothyroidism [119].

To further explore the mechanisms underlying this association, a 2021 review encompassing 45 studies provided additional insight into the potential biological links between psoriasis and AITD. Overall, most studies supported a positive association between the two conditions, although a minority failed to demonstrate a significant relationship, highlighting the heterogeneity of the available evidence. The analysis identified both genetic and immunological factors that may contribute to this association. Genetic susceptibility was supported by two studies reporting the involvement of long non-coding RNA (lncRNA) and polymorphisms of the signal transducer and activator of transcription 4 (STAT4) gene in both conditions. Additionally, six studies described shared immune pathways involving IL-17 and several chemokines, including CXCL10, CXCL9, CCL2, and CCL22, suggesting overlapping inflammatory processes underlying psoriasis and thyroid autoimmunity. Furthermore, elevated levels of adipokines, especially leptin, have been reported in both psoriasis and thyroid disorders and may contribute to the increased prevalence of anti-TPO antibodies observed in obese psoriatic patients. Finally, it has been reported that treatment with etanercept was associated with improvement of psoriatic skin lesions and changes in thyroid-related parameters, including a negative correlation between TNF-α and TSH levels, while untreated patients exhibited significantly higher FT4 concentrations. These findings suggest a potential interaction between TNF-α-mediated inflammatory pathways and thyroid function in patients with psoriasis [115].

### 4.2. Association of Psoriasis with Systemic Lupus Erythematosus

Systemic lupus erythematosus (SLE) is a multisystem autoimmune disease characterized by the production of antinuclear antibodies (ANAs), predominantly affecting young women. Among ANAs, anti-Smith (anti-Sm) and anti-double-stranded DNA (anti-dsDNA) antibodies are particularly characteristic of SLE. Cutaneous involvement represents one of the most common clinical features and is categorized into lupus-specific and lupus-nonspecific lesions according to their histopathological characteristics. Lupus-specific lesions include acute cutaneous lupus erythematosus (ACLE), subacute cutaneous lupus erythematosus (SCLE), and chronic cutaneous lupus erythematosus (CCLE) forms, while lupus-nonspecific manifestations include vasculitis, livedo reticularis and Raynaud’s phenomenon [120,121].

Although SLE and psoriasis have traditionally been regarded as distinct immune-mediated disorders, growing evidence suggests shared genetic and pathogenic mechanisms and potential clinical overlap, as both diseases may affect similar organ systems, particularly the cutaneous, musculoskeletal, renal, and cardiovascular systems, and may also coexist with secondary metabolic disorders. SLE and psoriasis share several key immunological mechanisms, including IFN-mediated signalling, activation of plasmacytoid dendritic cells, and immune responses triggered by DNA-LL-37 complexes, all of which contribute to the initiation and maintenance of chronic inflammation [14,122,123]. Further supporting a possible immunological link between these disorders, patients with psoriasis show a higher prevalence of circulating ANA than healthy individuals (44.3% vs. 11.5%), with ANA titers correlating with disease severity [124]. Nevertheless, the coexistence of SLE and psoriasis remains relatively uncommon, with an estimated prevalence of approximately 0.69% across reported studies [124]. Interestingly, a cohort study reported a proportionally higher representation of male patients among individuals with concomitant SLE and psoriasis. Additionally, patients with coexisting psoriasis reported a greater overall pain burden and more frequent methotrexate use, while no significant differences in clinical phenotypes or damage accrual were observed compared with patients without psoriasis [125].

Although phototherapy represents a cornerstone in the management of patients with psoriasis [126], UV radiation in SLE may act as a trigger for disease onset or exacerbations; therefore, photoprotection is recommended for all patients [127,128,129]. It should be noted, however, that through mechanisms that remain incompletely understood, a minority of patients with psoriasis may also experience disease exacerbation following photoexposure. Interestingly, the coexistence of SLE and psoriasis appears to be associated with a greater degree of photosensitivity compared with either condition alone [130]. At the same time, despite their established role in SLE management, antimalarial agents such as hydroxychloroquine and chloroquine may complicate treatment in patients with concomitant psoriasis, as worsening of pre-existing psoriasis and the development of psoriasiform skin lesions have been reported during treatment [131,132,133].

ACLE occurs in approximately 30–50% of patients with SLE and is classified into two forms: localized, represented by the malar rash, and generalized, characterized by the maculopapular rash of lupus. SCLE occurs in 10–15% of patients with SLE and includes two main subtypes: annular and papulosquamous (psoriasiform), with some patients presenting both forms simultaneously [121,134]. Although most cases are considered idiopathic, up to one-third of SCLE cases are drug-induced (DI-SCLE). DI-SCLE has been reported in association with several therapies used in psoriasis, including TNF-α inhibitors (etanercept, adalimumab, infliximab), IL-17 inhibitors (secukinumab), and IL-12/23 inhibitors (ustekinumab), as well as medications frequently prescribed for psoriasis-associated comorbidities, particularly antihypertensive agents, especially thiazide diuretics and calcium channel blockers [135,136,137,138,139,140]. DI-SCLE exhibits a serological profile similar to idiopathic SCLE, characterized by positive ANA, including specific subtypes such as anti-Ro/SS-A and anti-La/SS-B antibodies, which may serve as useful diagnostic markers in the differential diagnosis of psoriasiform lesions [140]. Finally, CCLE comprises several subtypes, with discoid lupus erythematosus (DLE) accounting for approximately 50% of cases [134].

Hair loss is a common manifestation of SLE, with nonscarring alopecia occurring in 40–70% of patients and often reflecting disease activity. In addition, “lupus hair,” characterized by fragile, broken hairs involving the frontal scalp, may occur during disease flares and represent a form of telogen effluvium. Careful assessment of hair loss is essential to distinguish lupus-associated alopecia from psoriatic scalp-related hair loss and other causes of alopecia [134].

Overall, despite the relatively uncommon coexistence of SLE and psoriasis, their clinical and immunological overlap may create important diagnostic and therapeutic challenges, highlighting the need for careful assessment and an individualized management approach.

### 4.3. Association of Psoriasis with Celiac Disease

Celiac disease (CD) is a chronic immune-mediated disorder triggered by the ingestion of gluten-containing cereals such as wheat, rye, and barley in genetically susceptible individuals, affecting about 1% of the population worldwide. In the intestinal lamina propria, gliadin peptides are deamidated by tissue transglutaminase 2 (TG2), generating deamidated gliadin peptides (DGPs). These peptides activate CD4+ T lymphocytes, leading to intestinal inflammation, the production of anti-DGP, anti-endomysium, and anti-TG2 antibodies, and ultimately villous atrophy of the small intestine [141]. Although the primary target organ is the small intestine, CD is a systemic immune-mediated disorder that may affect multiple organs, including the skin, where it can manifest as dermatitis herpetiformis [142].

Current evidence supports a significant bidirectional association between CD and psoriasis. A meta-analysis published in 2019 demonstrated that patients with psoriasis are approximately 2.16 times more likely to be diagnosed with CD, while individuals with CD are 1.8 times more likely to have psoriasis [143]. Genetic studies have identified overlapping susceptibility loci between CD and psoriasis, including a common polymorphism located near the IL-2 and IL-21 gene loci [144,145,146]. In addition, both disorders are characterized by T-cell-mediated immune responses, with evidence supporting the involvement of Th1, Th17, and γδ T cells in their pathogenesis [147]. Another important link is intestinal barrier dysfunction. In untreated or undiagnosed CD, increased intestinal permeability facilitates exposure to immunogenic triggers and may promote systemic immune activation [148]. Furthermore, gliadin exposure induces CD4+ T-cell responses and increased IFN-γ production, a cytokine that has been associated with psoriasis severity [149,150]. Interestingly, patients with psoriasis who have positive serological markers of gluten sensitivity have been shown to improve following a gluten-free diet, whereas similar benefits have not been observed in patients without such markers [151]. In addition, a lower prevalence of psoriasis has been reported among patients with celiac disease who adhered to a gluten-free diet for at least one year [152].

Taken together, the available evidence suggests that celiac disease and psoriasis share common genetic and immunological mechanisms. Recognition of this association is clinically relevant, as targeted screening for celiac disease should be considered in psoriasis patients presenting with gastrointestinal symptoms, iron-deficiency anemia, or a family history of celiac disease. Early identification of underlying CD may not only improve gastrointestinal outcomes but may also contribute to better management of psoriasis in selected patients.

## 5. Conclusions and Future Directions

Psoriasis is a complex immune-mediated condition associated with a broad spectrum of autoimmune diseases, although the strength of evidence supporting these associations varies among individual conditions.

The associations analyzed in this review involve both dermatological and systemic autoimmune conditions and may reflect shared genetic susceptibility loci and immunological pathways, with epidemiological data supporting a bidirectional relationship for some conditions.

Advances in understanding psoriasis immunopathogenesis, particularly the central role of the IL-23/Th17 axis, have supported the development of targeted treatments. Recent clinical and real-world evidence further supports the therapeutic relevance of these immunological mechanisms in the management of psoriatic disease.

The reported associations between psoriasis and autoimmune diseases are not entirely consistent across studies. This heterogeneity may be related to variability in study populations, demographic characteristics, diagnostic criteria, treatment exposure, adjustment for potential confounding factors, and study design, all of which may contribute to discrepancies in the reported prevalence and strength of these associations.

Recognition of these disorders is essential because their coexistence may influence clinical presentation, prognosis, therapeutic decisions, and most importantly, patient quality of life. Furthermore, several treatments used in the management of psoriasis have been temporally associated with the onset or exacerbation of autoimmune diseases, emphasizing the need for careful patient evaluation and individualized therapeutic strategies. Evidence regarding several treatment-associated autoimmune manifestations is based mainly on observational studies and individual reports, which do not establish a causal relationship.

Future research should focus on prospective studies to better characterize the temporal relationship between psoriasis and autoimmune disorders, identify biomarkers predictive of these associations, and evaluate the efficacy and safety of innovative treatments for the management of patients with coexisting immune-mediated diseases. A multidisciplinary approach remains the gold standard of care for these patients.

## Data Availability

No new data were created or analyzed in this study. Data sharing is not applicable to this article.

## References

[1] Griffiths C.E.M., Armstrong A.W., Gudjonsson J.E., Barker J.N.W.N. (2021). Psoriasis. Lancet.

[2] Mateescu L.A., Savu A.P., Mutu C.C., Vaida C.D., Șerban E.D., Bucur Ș., Poenaru E., Nicolescu A.-C., Constantin M.-M. (2024). The Intersection of Psoriasis and Neoplasia: Risk Factors, Therapeutic Approaches, and Management Strategies. Cancers.

[3] Bhagwat A.P., Madke B. (2023). The Current Advancement in Psoriasis. Cureus.

[4] Capon F. (2017). The Genetic Basis of Psoriasis. Int. J. Mol. Sci..

[5] Balak D.M., Hajdarbegovic E. (2017). Drug-induced psoriasis: Clinical perspectives. Psoriasis Targets Ther..

[6] Chandran V., Raychaudhuri S.P. (2010). Geoepidemiology and environmental factors of psoriasis and psoriatic arthritis. J. Autoimmun..

[7] Lupulescu A.M., Savu A.P., Bucur Ş., Şerban E.D., Popescu S., Constantin M.M. (2025). Hard-to-Treat Areas in Psoriasis: An Underevaluated Part of the Disease. Life.

[8] Rendon A., Schäkel K. (2019). Psoriasis Pathogenesis and Treatment. Int. J. Mol. Sci..

[9] Celoria V., Rosset F., Pala V., Dapavo P., Ribero S., Quaglino P., Mastorino L. (2023). The Skin Microbiome and Its Role in Psoriasis: A Review. Psoriasis Targets Ther..

[10] Ran D., Cai M., Zhang X. (2019). Genetics of psoriasis: A basis for precision medicine. Precis. Clin. Med..

[11] Patel H.A., Revankar R.R., Pedroza S.T., Graham S., Feldman S.R. (2023). The Genetic Susceptibility to Psoriasis and the Relationship of Linked Genes to Our Treatment Options. Int. J. Mol. Sci..

[12] Zhang M., Su W., Deng J., Zhai B., Zhu G., Gao R., Zeng Q., Qiu J., Bian Z., Xiao H. (2025). Multi-ancestry genome-wide meta-analysis with 472,819 individuals identifies 32 novel risk loci for psoriasis. J. Transl. Med..

[13] Nestle F.O., Conrad C., Tun-Kyi A., Homey B., Gombert M., Boyman O., Burg G., Liu Y.-J., Gilliet M. (2005). Plasmacytoid predendritic cells initiate psoriasis through interferon-α production. J. Exp. Med..

[14] Furue K., Ito T., Tsuji G., Kadono T., Nakahara T., Furue M. (2018). Autoimmunity and autoimmune co-morbidities in psoriasis. Immunology.

[15] Shirley S.N., Watson A.E., Yusuf N. (2024). Pathogenesis of Inflammation in Skin Disease: From Molecular Mechanisms to Pathology. Int. J. Mol. Sci..

[16] Hu P., Wang M., Gao H., Zheng A., Li J., Mu D., Tong J. (2021). The Role of Helper T Cells in Psoriasis. Front. Immunol..

[17] Qu N., Xu M., Mizoguchi I., Furusawa J., Kaneko K., Watanabe K., Mizuguchi J., Itoh M., Kawakami Y., Yoshimoto T. (2013). Pivotal Roles of T-Helper 17-Related Cytokines, IL-17, IL-22, and IL-23, in Inflammatory Diseases. Clin. Dev. Immunol..

[18] Korn T., Bettelli E., Oukka M., Kuchroo V.K. (2009). IL-17 and Th17 Cells. Annu. Rev. Immunol..

[19] Campanati A., Marani A., Martina E., Diotallevi F., Radi G., Offidani A. (2021). Psoriasis as an Immune-Mediated and Inflammatory Systemic Disease: From Pathophysiology to Novel Therapeutic Approaches. Biomedicines.

[20] Branisteanu D.E., Cojocaru C., Diaconu R., Porumb E.A., Alexa A.I., Nicolescu A.C., Brihan I., Bogdanici C.M., Branisteanu G., Dimitriu A. (2022). Update on the etiopathogenesis of psoriasis (Review). Exp. Ther. Med..

[21] Lande R., Botti E., Jandus C., Dojcinovic D., Fanelli G., Conrad C., Chamilos G., Feldmeyer L., Marinari B., Chon S. (2014). The antimicrobial peptide LL37 is a T-cell autoantigen in psoriasis. Nat. Commun..

[22] Arakawa A., Siewert K., Stöhr J., Besgen P., Kim S.M., Rühl G., Nickel J., Vollmer S., Thomas P., Krebs S. (2015). Melanocyte antigen triggers autoimmunity in human psoriasis. J. Exp. Med..

[23] Sieminska I., Pieniawska M., Grzywa T.M. (2024). The Immunology of Psoriasis—Current Concepts in Pathogenesis. Clin. Rev. Allergy Immunol..

[24] Potestio L., Martora F., Lauletta G., Vallone Y., Battista T., Megna M. (2024). The Role of Interleukin 23/17 Axis in Psoriasis Management: A Comprehensive Review of Clinical Trials. Clin. Cosmet. Investig. Dermatol..

[25] Conforti A., Ariani A., Gentile M., Cataldi G., Becciolini A., Marchesoni A., Parisi S., Addimanda O., Celletti E., Idolazzi L. (2026). Six months predictors of DAPSA Remission with Guselkumab in Psoriatic Arthritis in a Multicenter Real-World Study. Sci. Rep..

[26] Sticherling M. (2016). Psoriasis and autoimmunity. Autoimmun. Rev..

[27] Hakami A.R., AlSalman S.A., Aljaziri N.J., Homoud B., Almohideb M. (2024). Prevalence of Autoimmune Diseases Among Patients with Psoriasis: A Single Tertiary Center Experience. Cureus.

[28] Feng X., Zheng H., Wang M., Wang Y., Zhou X., Zhang X., Li J., Xiao Y., Wei M., Li X. (2025). Autoimmune bullous diseases: Pathogenesis and clinical management. Mol. Biomed..

[29] Didona D., Maglie R., Eming R., Hertl M. (2019). Pemphigus: Current and Future Therapeutic Strategies. Front. Immunol..

[30] Pisanti S., Sharav Y., Kaufman E., Posner L.N. (1974). Pemphigus vulgaris: Incidence in Jews of different ethnic groups, according to age, sex, and initial lesion. Oral Surg. Oral Med. Oral Pathol..

[31] Kridin K., Zelber-Sagi S., Bergman R. (2017). Pemphigus Vulgaris and Pemphigus Foliaceus: Differences in Epidemiology and Mortality. Acta Derm.-Venereol..

[32] Kridin K., Schmidt E. (2021). Epidemiology of Pemphigus. JID Innov..

[33] Tsai T.F., Wang T.S., Hung S.T., Tsai P.I.C., Schenkel B., Zhang M., Tang C.-H. (2011). Epidemiology and comorbidities of psoriasis patients in a national database in Taiwan. J. Dermatol. Sci..

[34] Kridin K., Ludwig R.J., Damiani G., Cohen A.D. (2020). Increased Risk of Pemphigus among Patients with Psoriasis: A Large-scale Cohort Study. Acta Derm.-Venereol..

[35] Ohata C., Ishii N., Koga H., Fukuda S., Tateishi C., Tsuruta D., Furumura M., Hashimoto T. (2015). Coexistence of autoimmune bullous diseases (AIBDs) and psoriasis: A series of 145 cases. J. Am. Acad. Dermatol..

[36] Dainichi T., Kabashima K. (2018). Interaction of Psoriasis and Bullous Diseases. Front. Med..

[37] Zhang X., Mao X., Theresia C., Xu R., Dong J., Pan M., Yuan H. (2018). Pemphigus Associated with Psoriasis Vulgaris: A Retrospective Study of Seven Patients and a Review of the Literature. Acta Dermatovenerol. Croat..

[38] Kridin K., Ludwig R.J. (2018). The Growing Incidence of Bullous Pemphigoid: Overview and Potential Explanations. Front. Med..

[39] Marazza G., Pham H.C., Schärer L., Pedrazzetti P.P., Hunziker T., Trüeb R.M., Hohl D., Itin P., Lautenschlager S., Naldi L. (2009). Incidence of bullous pemphigoid and pemphigus in Switzerland: A 2-year prospective study. Br. J. Dermatol..

[40] Joly P., Baricault S., Sparsa A., Bernard P., Bédane C., Duvert-Lehembre S., Courville P., Bravard P., Rémond B., Doffoel-Hantz V. (2012). Incidence and Mortality of Bullous Pemphigoid in France. J. Investig. Dermatol..

[41] Kridin K., Bergman R. (2018). Ethnic variations in the epidemiology of bullous pemphigoid in Israel. Int. J. Dermatol..

[42] Dainichi T., Koga H., Tsuji T., Ishii N., Ohyama B., Ueda A., Natsuaki Y., Karashima T., Nakama T., Yasumoto S. (2010). From anti-p200 pemphigoid to anti-laminin γ1 pemphigoid. J. Dermatol..

[43] Ho Y.H., Hu H.Y., Chang Y.T., Li C.P., Wu C.Y. (2019). Psoriasis is associated with increased risk of bullous pemphigoid: A nationwide population-based cohort study in Taiwan. J. Dermatol..

[44] Chen Y.J., Wu C.Y., Lin M.W., Chen T.J., Liao K.K., Chen Y.C., Hwang C., Chu S., Chen C., Lee D. (2011). Comorbidity profiles among patients with bullous pemphigoid: A nationwide population-based study. Br. J. Dermatol..

[45] Lazarczyk M., Wozniak K., Ishii N., Gorkiewicz-Petkov A., Hashimoto T., Schwarz R., Kowalewski C. (2006). Coexistence of psoriasis and pemphigoid-only a coincidence?. Int. J. Mol. Med..

[46] Kridin K., Bergman R. (2017). Association between bullous pemphigoid and psoriasis: A case-control study. J. Am. Acad. Dermatol..

[47] Loiseau P., Lecleach L., Prost C., Lepage V., Busson M., Bastuji-Garin S., Roujeaub J.C., Charrona D. (2000). HLA class II polymorphism contributes to specify desmoglein derived peptides in pemphigus vulgaris and pemphigus foliaceus. J. Autoimmun..

[48] Krueger J.G., Bowcock A. (2005). Psoriasis pathophysiology: Current concepts of pathogenesis. Ann. Rheum. Dis..

[49] Didona D., Di Zenzo G. (2018). Humoral Epitope Spreading in Autoimmune Bullous Diseases. Front. Immunol..

[50] Chan L.S., Vanderlugt C.J., Hashimoto T., Nishikawa T., Zone J.J., Black M.M., Wojnarowska F., Stevens S.R., Chen M., Fairley J.A. (1998). Epitope spreading: Lessons from autoimmune skin diseases. J. Investig. Dermatol..

[51] Maronese C.A., Cassano N., Genovese G., Foti C., Vena G.A., Marzano A.V. (2022). The Intriguing Links between Psoriasis and Bullous Pemphigoid. J. Clin. Med..

[52] Lee C.W., Ro Y.S. (1994). Pemphigus developed on preexisting dermatoses. J. Dermatol..

[53] Robati R.M., Faramarzirad F., Gheisari M., Zahedi K. (2025). Coexistence of Psoriasis and Pemphigus: A Case Report of Pemphigus Vulgaris with a Review of the Literature. Clin. Case Rep..

[54] Chen K.R., Shimizu S., Miyakawa S., Ishiko A., Shimizu H., Hashimoto T. (1996). Coexistence of psoriasis and an unusual IgG-mediated subepidermal bullous dermatosis: Identification of a novel 200-kDa lower lamina lucida target antigen. Br. J. Dermatol..

[55] Kobayashi T.T., Elston D.M., Libow L.F., David-Bajar K. (2002). A case of bullous pemphigoid limited to psoriatic plaques. Cutis.

[56] Grattan C.E. (1985). Evidence of an association between bullous pemphigoid and psoriasis. Br. J. Dermatol..

[57] Arakawa M., Dainichi T., Ishii N., Hamada T., Karashima T., Nakama T., Yasumoto S., Tsuruta D., Hashimoto T. (2011). Lesional Th17 cells and regulatory T cells in bullous pemphigoid. Exp. Dermatol..

[58] Yasukawa S., Dainichi T., Kokuba H., Moroi Y., Urabe K., Hashimoto T., Furue M. (2009). Bullous pemphigoid followed by pustular psoriasis showing Th1, Th2, Treg and Th17 immunological changes. Eur. J. Dermatol. EJD.

[59] Panzarella K., Camisa C. (1996). Coexistence of superficial pemphigus and psoriasis. Cutis.

[60] Verheyden M.J., Bilgic A., Murrell D.F. (2020). A Systematic Review of Drug-Induced Pemphigoid. Acta Derm.-Venereol..

[61] Yamazaki F. (2021). Psoriasis: Comorbidities. J. Dermatol..

[62] Husein-ElAhmed H., Steinhoff M. (2022). Bullous pemphigoid induced by biologic drugs in psoriasis: A systematic review. J. Dermatol. Treat..

[63] Stone C., Bak G., Oh D., Zhao C., Venugopal S., Kumar K., Murrell D.F. (2024). Environmental triggers of pemphigus vulgaris and bullous pemphigoid: A case control study. Front. Med..

[64] Caca-Biljanovska N., Arsovska-Bezhoska I., V’lckova-Laskoska M. (2016). PUVA-induced Bullous Pemphigoid in Psoriasis. Acta Dermatovenerol. Croat. ADC.

[65] Ezzedine K., Eleftheriadou V., Whitton M., van Geel N. (2015). Vitiligo. Lancet.

[66] Bergqvist C., Ezzedine K. (2020). Vitiligo: A Review. Dermatology.

[67] Taneja N., Sreenivas V., Sahni K., Gupta V., Ramam M. (2021). Disease Stability in Segmental and Non-Segmental Vitiligo. Indian Dermatol. Online J..

[68] Ezzedine K., Lim H.W., Suzuki T., Katayama I., Hamzavi I., Lan C.C.E., Goh B.K., Anbar T., de Castro C.S., Lee A.Y. (2012). Revised classification/nomenclature of vitiligo and related issues: The Vitiligo Global Issues Consensus Conference. Pigment Cell Melanoma Res..

[69] Li J., Shen Q., Wang Y., Guo C., Ma Y., Zhang Y. (2024). The relationship between psoriasis and vitiligo: From a comprehensive study. Ski. Res. Technol..

[70] Sawchuk M., Spano F., Loo W.J., Guenther L. (2012). The coexistence of psoriasis and vitiligo: A review. J. Cutan. Med. Surg..

[71] Sharquie K.E., Salman H.A., Yaseen A.K. (2017). Psoriasis and vitiligo are close relatives. Clin. Cosmet. Investig. Dermatol..

[72] Zhu K.J., Lv Y.M., Yin X.Y., Wang Z.X., Sun L.D., He S.M., Cheng H., Hu D.-Y., Zhang Z., Li Y. (2011). Psoriasis Regression Analysis of MHC Loci Identifies Shared Genetic Variants with Vitiligo. PLoS ONE.

[73] Wu J.J., Nguyen T.U., Poon K.Y.T., Herrinton L.J. (2012). The association of psoriasis with autoimmune diseases. J. Am. Acad. Dermatol..

[74] Augustin M., Radtke M.A., Glaeske G., Reich K., Christophers E., Schaefer I., Jacobi A. (2015). Epidemiology and Comorbidity in Children with Psoriasis and Atopic Eczema. Dermatology.

[75] Blegvad C., Egeberg A., Nielsen T., Gislason G., Zachariae C., Andersen A., Skov L. (2017). Autoimmune Disease in Children and Adolescents with Psoriasis: A Cross-sectional Study in Denmark. Acta Derm.-Venereol..

[76] Radtke M.A., Schäfer I., Glaeske G., Jacobi A., Augustin M. (2017). Prevalence and comorbidities in adults with psoriasis compared to atopic eczema. J. Eur. Acad. Dermatol. Venereol. JEADV.

[77] Zander N., Schäfer I., Radtke M., Jacobi A., Heigel H., Augustin M. (2017). Dermatological comorbidity in psoriasis: Results from a large-scale cohort of employees. Arch. Dermatol. Res..

[78] Lee H., Lee M.H., Lee D.Y., Kang H.Y., Kim K.H., Choi G.S., Shin J., Lee H.J., Kim D.H., Kim T.H. (2015). Prevalence of vitiligo and associated comorbidities in Korea. Yonsei Med. J..

[79] Teulings H.E., Ceylan E., Overkamp M., Vrijman C., Bos J.D., Nijsten T.E., Wolkerstorfer A., Luiten R., van der Veen J. (2016). Nonsegmental vitiligo disease duration and female sex are associated with comorbidity and disease extent: A retrospective analysis in 1307 patients aged ≥ 50 years. Br. J. Dermatol..

[80] Yen H., Chi C.C. (2019). Association Between Psoriasis and Vitiligo: A Systematic Review and Meta-Analysis. Am. J. Clin. Dermatol..

[81] Kridin K., Lyakhovitsky K., Onn E., Lyakhovitsky A., Ludwig R., Weinstein O., Cohen A.D. (2023). Investigating the epidemiological relationship between vitiligo and psoriasis: A population-based study. Arch. Dermatol. Res..

[82] Lim J.H., Lew B.L., Sim W.Y., Kwon S.H. (2022). Incidence of childhood-onset vitiligo and increased risk of atopic dermatitis, autoimmune diseases, and psoriasis: A nationwide population-based study. J. Am. Acad. Dermatol..

[83] Ji Y.Z., Liu S.R. (2019). Koebner phenomenon leading to the formation of new psoriatic lesions: Evidences and mechanisms. Biosci. Rep..

[84] Ono S., Tanizaki H., Otsuka A., Endo Y., Koyanagi I., Kataoka T.R., Miyachi Y., Kabashima K. (2014). Coexistent skin lesions of vitiligo and psoriasis vulgaris. Immunohistochemical analyses for IL-17A-producing cells and regulatory T cells. Acta Derm.-Venereol..

[85] Aghamajidi A., Raoufi E., Parsamanesh G., Jalili A., Salehi-Shadkami M., Mehrali M., Mohsenzadegan M. (2021). The attentive focus on T cell-mediated autoimmune pathogenesis of psoriasis, lichen planus and vitiligo. Scand. J. Immunol..

[86] Das D., Akhtar S., Kurra S., Gupta S., Sharma A. (2019). Emerging role of immune cell network in autoimmune skin disorders: An update on pemphigus, vitiligo and psoriasis. Cytokine Growth Factor. Rev..

[87] Simsek H., Savas C., Akkiz H., Telatar H. (1996). Interferon-induced vitiligo in a patient with chronic viral hepatitis C infection. Dermatology.

[88] Hamadah I., Binamer Y., Sanai F.M., Abdo A.A., Alajlan A. (2010). Interferon-induced vitiligo in hepatitis C patients: A case series. Int. J. Dermatol..

[89] Afshar M., Martinez A.D., Gallo R.L., Hata T.R. (2013). Induction and exacerbation of psoriasis with Interferon-alpha therapy for hepatitis C: A review and analysis of 36 cases. J. Eur. Acad. Dermatol. Venereol..

[90] Fattovich G., Giustina G., Favarato S., Ruol A. (1996). A survey of adverse events in 11,241 patients with chronic viral hepatitis treated with alfa interferon. J. Hepatol..

[91] Damsky W., King B.A. (2017). JAK inhibitors in dermatology: The promise of a new drug class. J. Am. Acad. Dermatol..

[92] Kuo C.M., Tung T.H., Wang S.H., Chi C.C. (2018). Efficacy and safety of tofacitinib for moderate-to-severe plaque psoriasis: A systematic review and meta-analysis of randomized controlled trials. J. Eur. Acad. Dermatol. Venereol..

[93] Malerba M., Damiani G., Radaeli A., Ragnoli B., Olivini A., Calzavara-Pinton P.G. (2015). Narrowband ultraviolet B phototherapy in psoriasis reduces proinflammatory cytokine levels and improves vitiligo and neutrophilic asthma. Br. J. Dermatol..

[94] Burlando M., Muracchioli A., Cozzani E., Parodi A. (2021). Psoriasis, Vitiligo, and Biologic Therapy: Case Report and Narrative Review. Case Rep. Dermatol..

[95] Bae J.M., Kim M., Lee H.H., Kim K.J., Shin H., Ju H.J., Kim G.M., Park C.J., Park H.J. (2018). Increased Risk of Vitiligo Following Anti-Tumor Necrosis Factor Therapy: A 10-Year Population-Based Cohort Study. J. Investig. Dermatol..

[96] Sibbald C. (2023). Alopecia Areata: An Updated Review for 2023. J. Cutan. Med. Surg..

[97] Gilhar A., Etzioni A., Paus R. (2012). Alopecia areata. N. Engl. J. Med..

[98] Zhou C., Li X., Wang C., Zhang J. (2021). Alopecia Areata: An Update on Etiopathogenesis, Diagnosis, and Management. Clin. Rev. Allergy Immunol..

[99] Lee H.H., Gwillim E., Patel K.R., Hua T., Rastogi S., Ibler E., Silverberg J.I. (2020). Epidemiology of alopecia areata, ophiasis, totalis, and universalis: A systematic review and meta-analysis. J. Am. Acad. Dermatol..

[100] Wu M.C., Yang C.C., Tsai R.Y., Chen W.C. (2013). Late-onset alopecia areata: A retrospective study of 73 patients from Taiwan. J. Eur. Acad. Dermatol. Venereol..

[101] Jung J.M., Yang H.J., Lee W.J., Won C.H., Lee M.W., Chang S.E. (2022). Association between psoriasis and alopecia areata: A systematic review and meta-analysis. J. Dermatol..

[102] Villasante Fricke A.C., Miteva M. (2015). Epidemiology and burden of alopecia areata: A systematic review. Clin. Cosmet. Investig. Dermatol..

[103] Elder J.T. (2013). What can the genetics of psoriasis teach us about alopecia areata?. J. Investig. Dermatol. Symp. Proc..

[104] Perez S.M., Kabashima K., Reich K., Gilliet M., Gilhar A., Paus R. (2025). Lessons from TNF-α Inhibitor-Induced Alopecia Areata: Does TNF-α Operate as a Hair Follicle Immune Privilege Guardian under Inflammatory Conditions?. J. Investig. Dermatol..

[105] Kennedy Crispin M., Ko J.M., Craiglow B.G., Li S., Shankar G., Urban J.R., Chen J.C., Cerise J.E., Jabbari A., Winge M.C. (2016). Safety and efficacy of the JAK inhibitor tofacitinib citrate in patients with alopecia areata. JCI Insight.

[106] Słuczanowska-Głąbowska S., Ziegler-Krawczyk A., Szumilas K., Pawlik A. (2021). Role of Janus Kinase Inhibitors in Therapy of Psoriasis. J. Clin. Med..

[107] Schons K.R.R., Knob C.F., Murussi N., Beber A.A.C., Neumaier W., Monticielo O.A. (2014). Nail psoriasis: A review of the literature. An. Bras. Dermatol..

[108] Pourani M.R., Khajeamiri Y., Abdollahimajd F., Zargari O. (2025). Psoriasis and Alopecia: Unveiling the Links. Dermatol. Pract. Concept..

[109] Petranović Ovčariček P., Görges R., Giovanella L. (2024). Autoimmune Thyroid Diseases. Semin. Nucl. Med..

[110] Chistiakov D.A. (2005). Immunogenetics of Hashimoto’s thyroiditis. J. Autoimmune Dis..

[111] Wang C., Crapo L.M. (1997). The epidemiology of thyroid disease and implications for screening. Endocrinol. Metab. Clin..

[112] Subekti I., Pramono L.A. (2018). Current Diagnosis and Management of Graves’ Disease. Acta Medica Indones..

[113] Cira C.I., Carsote M., Nistor C., Petca A., Petca R.C., Sandru F. (2023). Conundrum for Psoriasis and Thyroid Involvement. Int. J. Mol. Sci..

[114] Hofman A., Brusselle G.G.O., Darwish Murad S., van Duijn C.M., Franco O.H., Goedegebure A., Ikram M.A., Klaver C.C.W., Nijsten T.E.C., Peeters R.P. (2015). The Rotterdam Study: 2016 objectives and design update. Eur. J. Epidemiol..

[115] Eapi S., Chowdhury R., Lawal O.S., Mathur N., Malik B.H. (2021). Etiological Association Between Psoriasis and Thyroid Diseases. Cureus.

[116] Khan S.R., Bano A., Wakkee M., Korevaar T.I.M., Franco O.H., Nijsten T.E.C., Peeters R.P., Chaker L. (2017). The association of autoimmune thyroid disease (AITD) with psoriatic disease: A prospective cohort study, systematic review and meta-analysis. Eur. J. Endocrinol..

[117] Zhang X., Zhang S., Wu R., Li S., Su Y., Zhang P. (2022). Prevalence of autoimmune thyroid disease in patients with psoriasis: A meta-analysis. BMJ Open.

[118] Alidrisi H.A., Al Hamdi K., Mansour A.A. (2019). Is There Any Association Between Psoriasis and Hashimoto’s Thyroiditis?. Cureus.

[119] Kwinter J., Weinstein M., Bargman H. (2007). Psoriasiform lesions and abscesses as initial manifestations of severe hypothyroidism in a previously healthy 15-year-old girl. Pediatr. Dermatol..

[120] Kaul A., Gordon C., Crow M.K., Touma Z., Urowitz M.B., van Vollenhoven R., Ruiz-Irastorza G., Hughes G. (2016). Systemic lupus erythematosus. Nat. Rev. Dis. Primer.

[121] Stull C., Sprow G., Werth V.P. (2023). Cutaneous Involvement in Systemic Lupus Erythematosus: A Review for the Rheumatologist. J. Rheumatol..

[122] Dai H., He F., Tsokos G.C., Kyttaris V.C. (2017). IL-23 Limits the Production of IL-2 and Promotes Autoimmunity in Lupus. J. Immunol..

[123] Garcia-Romo G.S., Caielli S., Vega B., Connolly J., Allantaz F., Xu Z., Punaro M., Baisch J., Guiducci C., Coffman R.L. (2011). Netting neutrophils are major inducers of type I IFN production in pediatric systemic lupus erythematosus. Sci. Transl. Med..

[124] Fijałkowska A., Wojtania J., Woźniacka A., Robak E. (2024). Psoriasis and Lupus Erythematosus-Similarities and Differences Between Two Autoimmune Diseases. J. Clin. Med..

[125] Walhelm T., Parodis I., Enerbäck C., Arkema E., Sjöwall C. (2025). Comorbid psoriasis in systemic lupus erythematosus: A cohort study from a tertiary referral centre and the National Patient Register in Sweden. Lupus Sci. Med..

[126] Rutter K.J., Watson R.E.B., Cotterell L.F., Brenn T., Griffiths C.E.M., Rhodes L.E. (2009). Severely photosensitive psoriasis: A phenotypically defined patient subset. J. Investig. Dermatol..

[127] Dowdy M.J., Nigra T.P., Barth W.F. (1989). Subacute cutaneous lupus erythematosus during PUVA therapy for psoriasis: Case report and review of the literature. Arthritis Rheum..

[128] Kulick K.B., Mogavero H., Provost T.T., Reichlin M. (1983). Serologic studies in patients with lupus erythematosus and psoriasis. J. Am. Acad. Dermatol..

[129] Yamamoto T. (2020). Psoriasis and Connective Tissue Diseases. Int. J. Mol. Sci..

[130] Hays S.B., Camisa C., Luzar M.J. (1984). The coexistence of systemic lupus erythematosus and psoriasis. J. Am. Acad. Dermatol..

[131] Kalia S., Dutz J.P. (2007). New concepts in antimalarial use and mode of action in dermatology. Dermatol. Ther..

[132] Gunawan H., Awalia A., Soeroso J. (2018). The Coexistence of Systemic Lupus Erythematosus and Psoriasis: Is It Possible?. Acta Medica Indones..

[133] Qu Y., Li D., Liu W., Shi D. (2023). Molecular consideration relevant to the mechanism of the comorbidity between psoriasis and systemic lupus erythematosus (Review). Exp. Ther. Med..

[134] Yell J.A., Mbuagbaw J., Burge S.M. (1996). Cutaneous manifestations of systemic lupus erythematosus. Br. J. Dermatol..

[135] Tarazi M., Aiempanakit K., Werth V.P. (2018). Subacute cutaneous lupus erythematosus and systemic lupus erythematosus associated with abatacept. JAAD Case Rep..

[136] Laurinaviciene R., Sandholdt L.H., Bygum A. (2017). Drug-induced cutaneous lupus erythematosus: 88 new cases. Eur. J. Dermatol..

[137] Keyes E., Grinnell M., Vazquez T., Diaz D., Thomas P., Werth V.P. (2021). Drug-induced subacute cutaneous lupus erythematosus in previously diagnosed systemic lupus erythematosus patients: A case series. JAAD Case Rep..

[138] Conforti C., Retrosi C., Giuffrida R., Vezzoni R., Currado D., Navarini L., Farinazzo E., Dell’Aquila C., Nan K., Di Meo N. (2020). Secukinumab-induced subacute cutaneous lupus erythematosus. Dermatol. Ther..

[139] Tierney E., Kirthi S., Ramsay B., Ahmad K. (2019). Ustekinumab-induced subacute cutaneous lupus. JAAD Case Rep..

[140] Borucki R., Werth V.P. (2020). Cutaneous lupus erythematosus induced by drugs—Novel insights. Expert. Rev. Clin. Pharmacol..

[141] Doyle J.B., Silvester J., Ludvigsson J.F., Lebwohl B. (2025). Advances in the pathophysiology, diagnosis, and management of celiac disease. BMJ.

[142] Reunala T., Salmi T.T., Hervonen K., Kaukinen K., Collin P. (2018). Dermatitis Herpetiformis: A Common Extraintestinal Manifestation of Coeliac Disease. Nutrients.

[143] Acharya P., Mathur M. (2020). Association between psoriasis and celiac disease: A systematic review and meta-analysis. J. Am. Acad. Dermatol..

[144] Tsoi L.C., Spain S.L., Knight J., Ellinghaus E., Stuart P.E., Capon F., Ding J., Li Y., Tejasvi T., Gudjonsson J.E. (2012). Identification of 15 new psoriasis susceptibility loci highlights the role of innate immunity. Nat. Genet..

[145] Einarsdottir E., Koskinen L.L.E., de Kauwe A.L., Dukes E., Mustalahti K., Balogh M., Korponay-Szabo I.R., Kaukinen K., Kurppa K., Ádány R. (2011). Genome-wide analysis of extended pedigrees confirms IL2-IL21 linkage and shows additional regions of interest potentially influencing coeliac disease risk. Tissue Antigens.

[146] Liu Y., Helms C., Liao W., Zaba L.C., Duan S., Gardner J., Wise C., Miner A., Malloy M.J., Pullinger C.R. (2008). A genome-wide association study of psoriasis and psoriatic arthritis identifies new disease loci. PLoS Genet..

[147] Bhatia B.K., Millsop J.W., Debbaneh M., Koo J., Linos E., Liao W. (2014). Diet and psoriasis, part II: Celiac disease and role of a gluten-free diet. J. Am. Acad. Dermatol..

[148] Ventura A., Magazzù G., Greco L. (1999). Duration of exposure to gluten and risk for autoimmune disorders in patients with celiac disease. SIGEP Study Group. for Autoimmune Disorders in Celiac Disease. Gastroenterology.

[149] Nilsen E.M., Jahnsen F.L., Lundin K.E., Johansen F.E., Fausa O., Sollid L.M., Jahnsen J., Scott H., Brandtzaeg P. (1998). Gluten induces an intestinal cytokine response strongly dominated by interferon gamma in patients with celiac disease. Gastroenterology.

[150] Abdallah M.A., Abdel-Hamid M.F., Kotb A.M., Mabrouk E.A. (2009). Serum interferon-gamma is a psoriasis severity and prognostic marker. Cutis.

[151] Michaëlsson G., Gerdén B., Hagforsen E., Nilsson B., Pihl-Lundin I., Kraaz W., Hjelmquist G., Lööf L. (2000). Psoriasis patients with antibodies to gliadin can be improved by a gluten-free diet. Br. J. Dermatol..

[152] Sategna Guidetti C., Solerio E., Scaglione N., Aimo G., Mengozzi G. (2001). Duration of gluten exposure in adult coeliac disease does not correlate with the risk for autoimmune disorders. Gut.

